# Supplementary material for: Transcriptome Analysis Reveals Putative Induction of Floral Initiation by Old Leaves in Tea-Oil Tree (Camellia oleifera ‘changlin53’)
Source: Int J Mol Sci. 2022 Oct 27;23(21):13021. doi: 10.3390/ijms232113021 (PMC9655362; doi:10.3390/ijms232113021)
Supplement: Supplementary file 1 [file ijms-23-13021-s001.zip › Supplementary Figures.pdf]

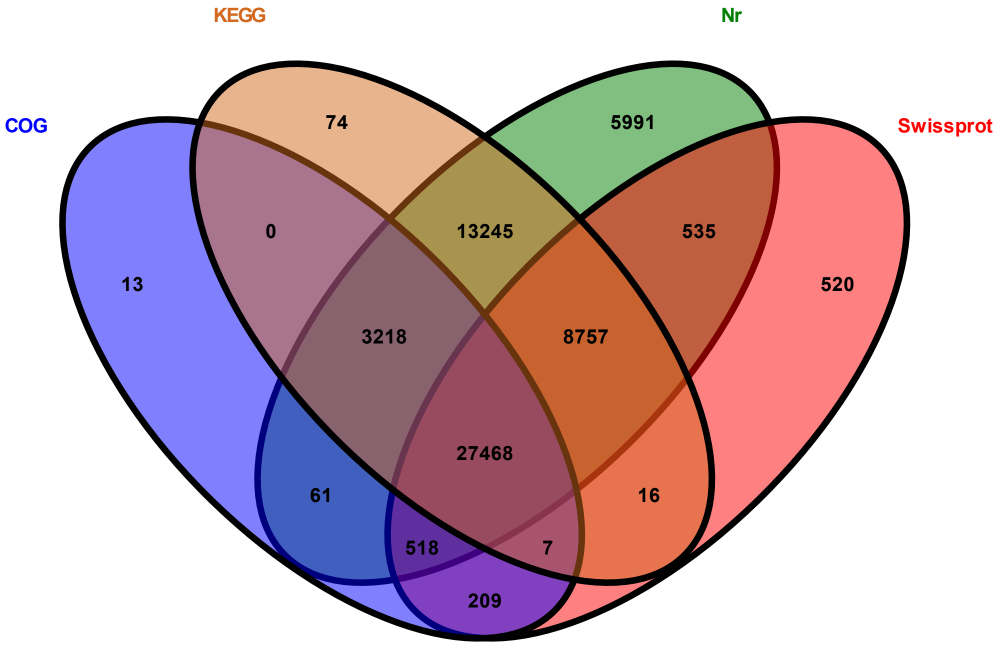

Figure S1. Venn diagram of unigenes annotated in COG, KEGG, Nr and Swissport databases.

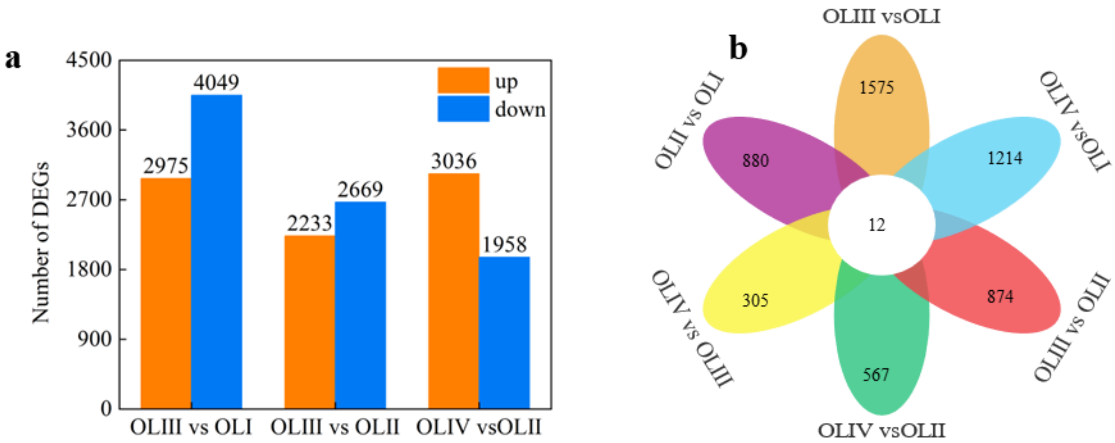

Figure S2. Numbers of DEGs in diverse pairwise comparisons (a) and venn diagram showing showing the common and unique DEGs among six different pairwise comparisons (b). The detailed information on the significantly DEGs of OLIII vs OLI, OLIV vs OLI and OLIV vs OLII is presented in Tables S4-S6.

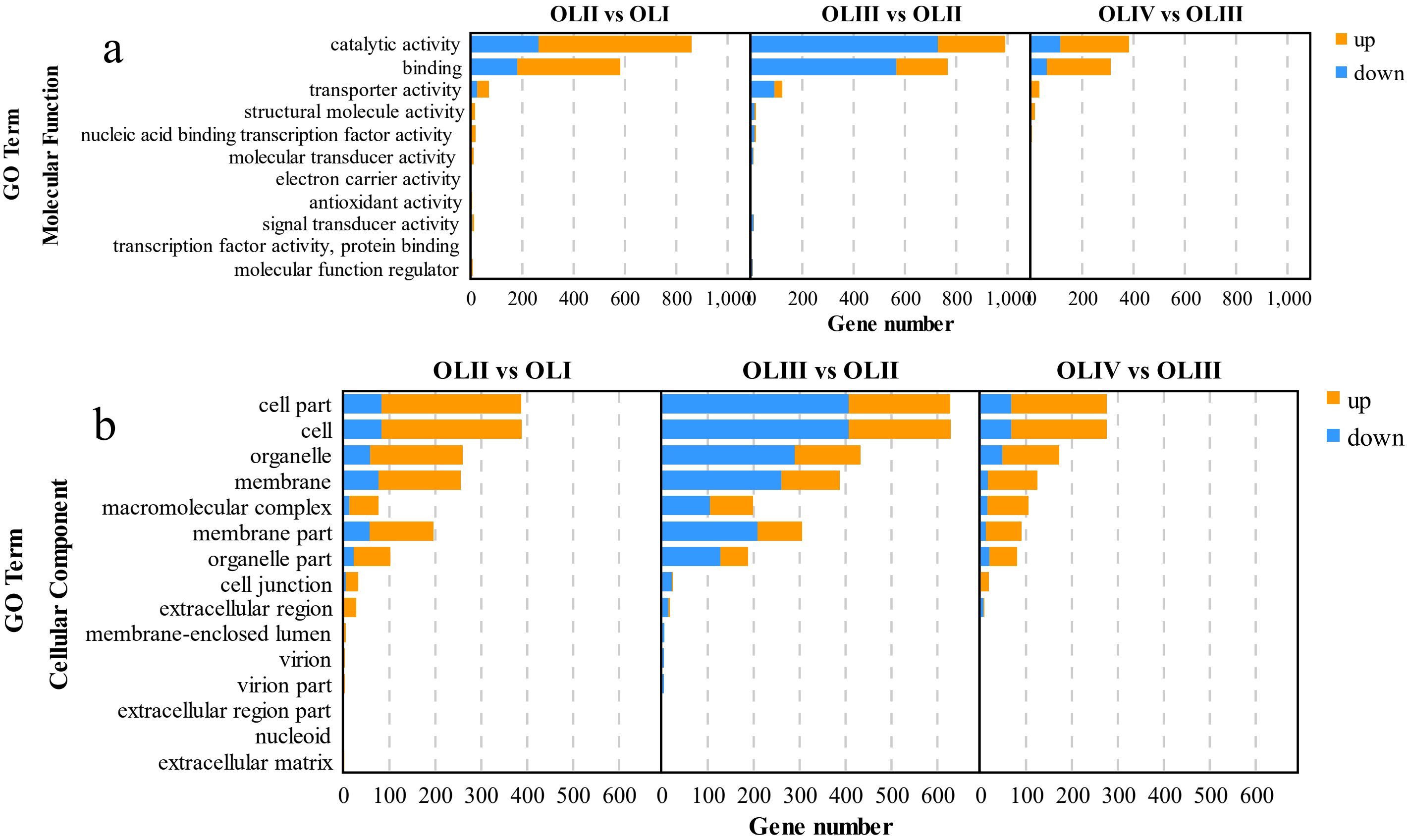

Figure S3. GO enrichment analysis of DEGs from old leaves. (a) Molecular function. (b) Cellular component.

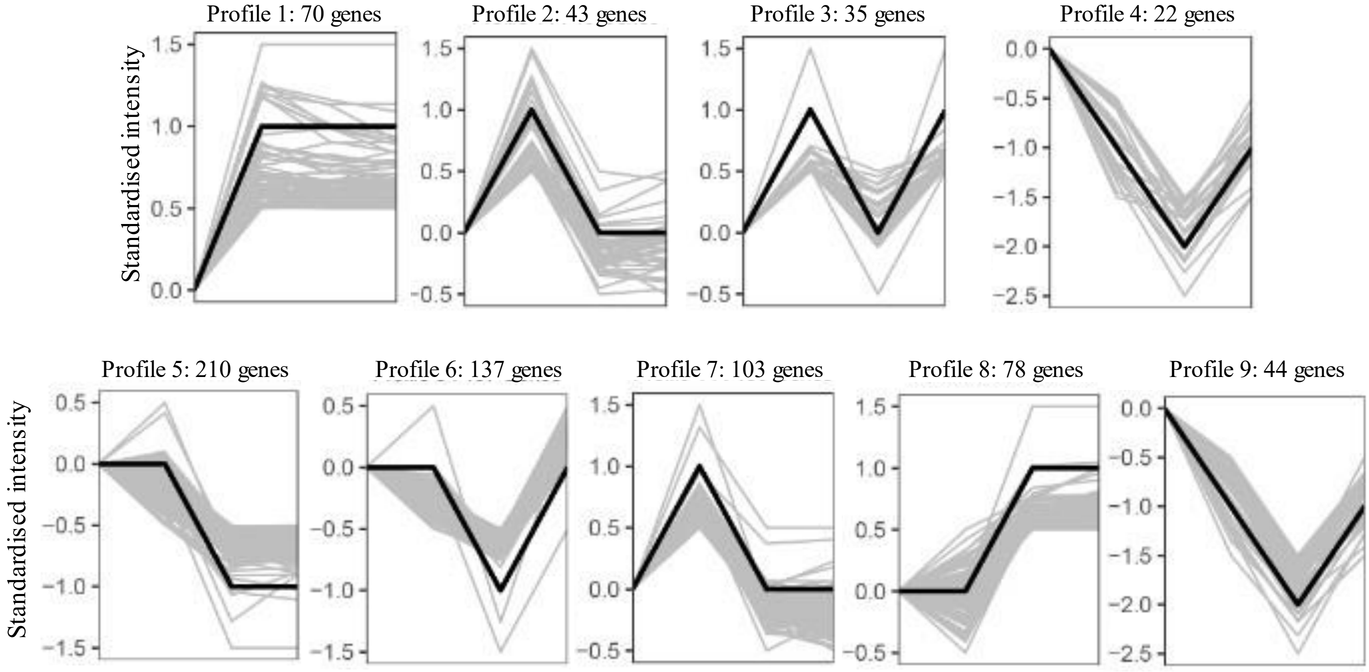

Figure S4. Profile 1-4 showed the significant expression patterns of DEGs associated with death in comparison OLII vs OLI. Profile 5-9 showed the significant expression patterns of DEGs associated with stimulus in comparison OLIII vs OLII.

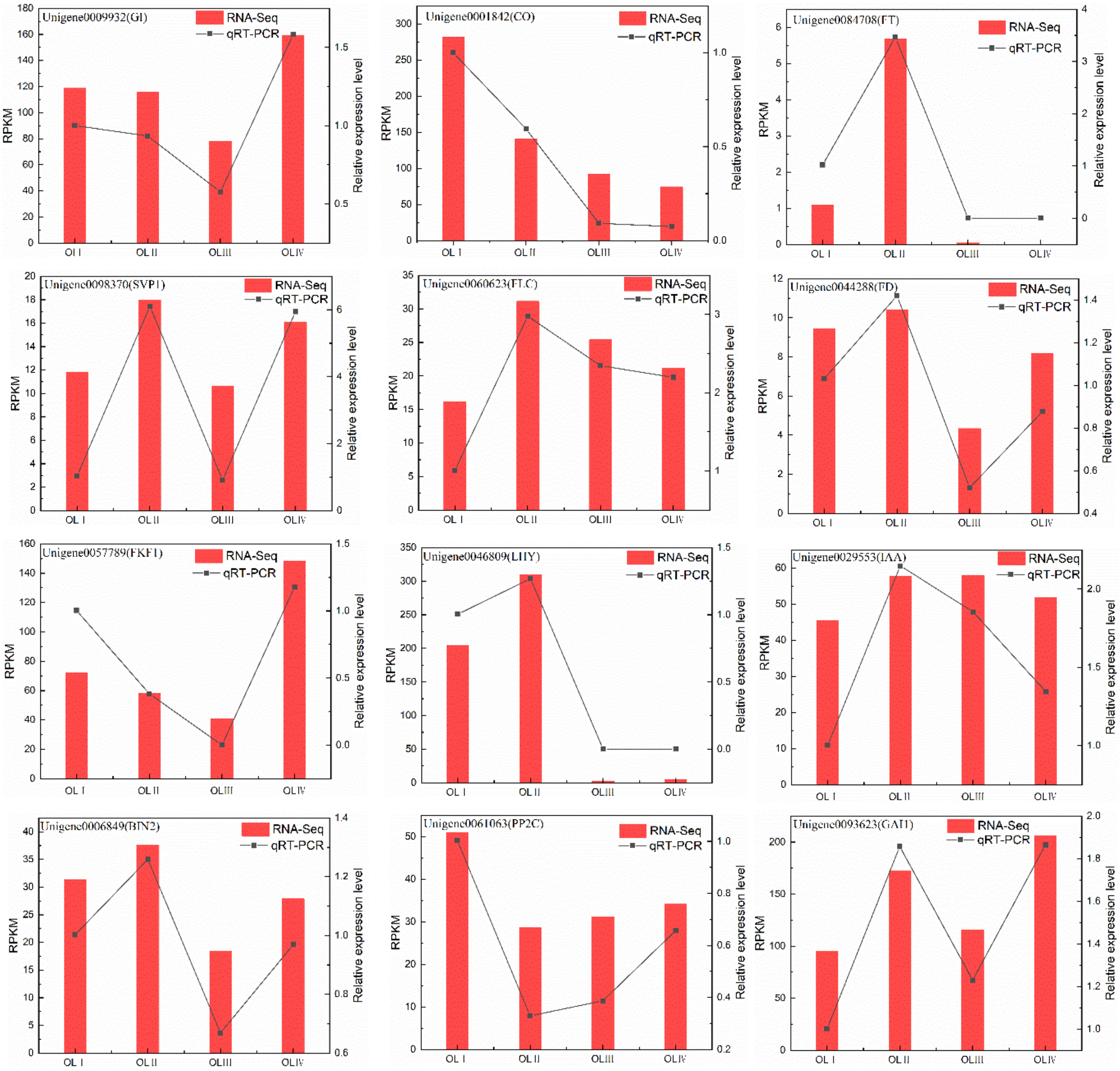

Figure S5. The comparison of the expression levels of 12 DEGs identified related to flowering, rhythm and hormone between RNA-Seq and qRT-PCR analyses from old leaves at different developmental stages during floral transition, *CoGAPDH* is used as internal control.
